# Robotic-assisted thoracoscopy in the management of mediastinal ectopic parathyroid adenoma: a case report

**DOI:** 10.1093/jscr/rjae044

**Published:** 2024-02-14

**Authors:** Kira C Steinkraus, Matthias Wittau, Marko Kornmann

**Affiliations:** Department of General and Visceral Surgery, University Hospital Ulm, Albert-Einstein-Allee 23, 89081 Ulm, Germany; Department of General and Visceral Surgery, University Hospital Ulm, Albert-Einstein-Allee 23, 89081 Ulm, Germany; Department of General and Visceral Surgery, University Hospital Ulm, Albert-Einstein-Allee 23, 89081 Ulm, Germany

**Keywords:** robotic mediastinal parathyroidectomy, primary hyperparathyroidism

## Abstract

Mediastinal ectopic parathyroid adenomas, a rare cause of primary hyperparathyroidism, has evolved significantly with the advent of robotic-assisted surgery. Traditional surgical approaches, while effective, may be associated with considerable morbidity and extended recovery periods. This study aims to evaluate the effectiveness, precision, and postoperative outcomes of robotic thoracoscopy with parathyroidectomy in the management of mediastinal ectopic parathyroid adenomas. A case of a 70-year-old man with a history of primary hyperparathyroidism underwent a successful left robotic thoracoscopy with parathyroidectomy in an ectopic mediastinal parathyroid adenoma. The robotic approach demonstrated advantages such as enhanced precision and minimal invasiveness. However, the learning curve and cost implications of this technology were identified as considerations. Robotic thoracoscopy with parathyroidectomy underscores the potential of robotic surgery in revolutionizing the management of mediastinal ectopic parathyroid adenomas, offering promising precision, emphasizing the need for ongoing research, and evaluation to optimize this innovative surgical method.

## Introduction

The ectopic parathyroid adenoma significantly contributes to persistent and recurring hyperparathyroidism. It is reported that up to 20% of ectopic parathyroid adenoma cases involve mediastinal placement of the ectopic tissue. In ~2% of instances, when a cervical approach is impractical, accessing the adenoma via the mediastinal route presents a surgical challenge [1, 2].

The management of mediastinal ectopic parathyroid adenomas, a rare but significant cause of primary hyperparathyroidism, has witnessed remarkable advancements in recent years. These adenomas, aberrantly located within the mediastinum, are responsible for the overproduction of parathyroid hormone (PTH), leading to hypercalcemia and its associated complications [3, 4]. Traditional surgical approaches, such as sternotomy and thoracotomy, have been employed to access and excise these ectopic glands but are often associated with considerable morbidity and extended recovery periods [1, 5].

In the quest for precision, minimal invasiveness, and enhanced postoperative outcomes, robotic-assisted surgery has emerged as a groundbreaking innovation in the realm of endocrine surgery. Robotic parathyroidectomy, leveraging state-of-the-art robotic systems, offers unparalleled advantages including superior three-dimensional visualization, enhanced dexterity, and the ability to navigate intricate anatomical spaces with remarkable precision [6–11]. This approach aims to offer patients a less invasive alternative, with the potential for reduced postoperative pain, quicker recovery times, and minimal scarring [12–14].

## Case report

**Figure 1:**
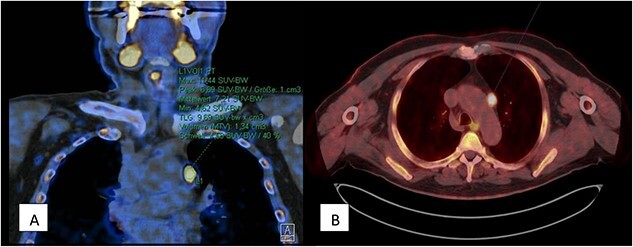
567 MBq C-11-Methionin (A) coronary view of upper mediastinum, (B) axial view with oval lesion with increased amino acid metabolism left of the aortic arch.

A 70-year-old man with a body mass index (BMI) of 32.6 presented himself 2021 with a history of primary hyperparathyroidism and a subtotal resection of a goiter in 2003. In January 2021, his PTH level was 129 pg/ml and calcium level was 2.78 mmol/l. An external parathyroid scintigraphy in April 2021 did not conclusively detect a parathyroid adenoma. For further diagnostics a 567 MBq C-11-Methionin was carried out in July 2021. Here in the upper mediastinum, to the left of the aortic arch, an oval lesion showed increased amino acid metabolism, raising suspicion of an ectopically located parathyroid adenoma (Fig. 1). Due to difficulty access caused by mediastinal localization we indicated the robotic- assisted approach in order to perform the parathyroidectomy.

The procedure began with the insertion of an 8 mm trocar into the left thoracic cavity after deflating the left lung, in the 5th intercostal space along the posterior axillary line. A camera was introduced, revealing no signs of injury upon entering the thoracic cavity. The lung collapsed well and the patient was further tilted to the right and in an anti-Trendelenburg position so that the lung moved downward and forward, making the descending aorta and the aortic arch clearly visible. Three more 8 mm trocars were placed: two intercostal spaces above the first, and two intercostal spaces below for the third and fourth. A 12 mm auxiliary trocar was placed between the second and third trocars. The robot (DaVinci Xi Surgical System® Intuitive Surgical Inc., Sunnyvale, CA, USA) was then docked and the target region was adjusted. The bipolar forceps were introduced through the first trocar, the camera through the second, scissors through the third, and holding forceps through the fourth.

The lung was moved aside and the aortic arch was inspected. A brownish lesion ~2 cm in diameter, the resumed adenoma, was identified at the same location indicated by the ´567 MBq C-11-Methionin scan. The parietal pleura was incised with scissors and the adenoma was carefully dissected out. Smaller blood vessels were sealed bipolarly and then cut. A slightly larger vessel leading to the aortic arch was clamped with metal clips and then cut. The adenoma was deposited in a specimen pouch. The parietal pleura was closed with a continuous absorbable suture (Stratafix 4.0) after ensuring hemostasis. The specimen was retrieved through the 12 mm trocar. A 20-gage straight thoracic drain was inserted under visual guidance through the same trocar. The lung was re-inflated after the removal of the instruments and expanded well. The trocars were removed and the wounds were closed with subcutaneous single-button sutures (Vicryl 3.0) and absorbable intracutaneous sutures . Blood loss was minimal.

During surgery, a significant drop in PTH levels was observed. The postoperative course was uneventful. The wounds were healing primarily and without irritation. The patient did not complain of tingling sensations at any time, and the postoperative calcium and PTH levels were within normal ranges and the patient was discharged in good general condition on postoperative day 3. Histopathology confirmed the presence of a parathyroid adenoma without malignant growth.

## Discussion

The introduction of robotic thoracoscopy marks a significant advancement in surgical techniques, especially for complex procedures like mediastinal ectopic parathyroidectomy [15–19]. This innovative approach diverges from traditional methods, offering a minimally invasive yet highly precise alternative. The benefits of this technique include faster recovery, minimal scarring, and reduced risk of complications, such as damage to adjacent structures like the recurrent laryngeal nerve and the thyroid gland.

Primary hyperparathyroidism, typically stemming from an adenoma in the parathyroid glands, has historically been addressed through conventional surgical techniques such as open surgery or standard minimally invasive procedures [20]. These traditional methods, while effective, may sometimes entail longer recovery periods, visible scarring, and a greater degree of postoperative discomfort. In this context, the application of robotic thoracoscopy provides an innovative solution as it is eminent for its ability to enhance surgical precision, stability, and reach, and its application in endocrine surgery is indeed groundbreaking [21, 22]. By employing a left robotic thoracoscopy for parathyroidectomy, surgeons can capitalize on enhanced dexterity and superior visualization afforded by the robotic system. The articulated instruments and high-definition three-dimensional vision system allow for a nuanced navigation and manipulation of tissues, which is particularly beneficial in the compact and delicate anatomical environment of the parathyroid glands.

However, the adoption of robotic techniques comes with challenges, including a learning curve for surgeons and significant cost implications. The expenses for materials and equipment in robotic surgery are notably higher than those for traditional laparoscopic surgery. In many healthcare systems, the reimbursement for robotic procedures may not be higher than that for laparoscopic surgeries, posing a financial challenge for healthcare providers. Balancing the advanced, patient-friendly surgical options with economic feasibility is crucial, especially when reimbursement rates do not align with the higher costs.

In conclusion, the successful implementation of robotic thoracoscopy for mediastinal ectopic parathyroidectomy showcases the revolutionary potential of robotic surgery in endocrine practices. With this case report we support the current interest in robotic surgery for endocrine surgery. Embracing these innovations could lead to enhanced patient outcomes and refined surgical methodologies, however, for the time being, healthcare providers need to carefully consider how to balance the benefits of robotic surgery with its higher costs.

Continuous research, evaluation, and dialog are essential to validate and optimize the utility of this approach in a broader spectrum of cases.
